# Differences in Early Stages of Tactile ERP Temporal Sequence (P100) in Cortical Organization during Passive Tactile Stimulation in Children with Blindness and Controls

**DOI:** 10.1371/journal.pone.0124527

**Published:** 2015-07-30

**Authors:** Tomás Ortiz Alonso, Juan Matías Santos, Laura Ortiz Terán, Mayelin Borrego Hernández, Joaquín Poch Broto, Gabriel Alejandro de Erausquin

**Affiliations:** 1 Department of Psychiatry, Facultad de Medicina, Universidad Complutense, Madrid, Spain; 2 Department of Psychology, Universidad de Atacama, Copiapó, Chile and Fundación J Robert Cade/CONICET, Córdoba, Argentina; 3 Athinoula A Martinos Center, Department of Radiology, Massachusetts General Hospital, Harvard University, Boston, Massachusetts, United States of America; 4 Centro de Neurociencias de Cuba, Habana, Cuba; 5 Department of Ear, Nose and Throat (ENT), Hospital Clínico Universitario San Carlos, Universidad Complutense, Madrid, Spain; 6 Center for Neuromodulation and Roskamp Laboratory of Brain Development, Modulation and Repair, Departments of Psychiatry, Neurology and Neurosurgery, University of South Florida, Tampa, Florida, United States of America; Liaoning Normal University, CHINA

## Abstract

Compared to their seeing counterparts, people with blindness have a greater tactile capacity. Differences in the physiology of object recognition between people with blindness and seeing people have been well documented, but not when tactile stimuli require semantic processing. We used a passive vibrotactile device to focus on the differences in spatial brain processing evaluated with event related potentials (ERP) in children with blindness (n = 12) vs. normally seeing children (n = 12), when learning a simple spatial task (lines with different orientations) or a task involving recognition of letters, to describe the early stages of its temporal sequence (from 80 to 220 msec) and to search for evidence of multi-modal cortical organization. We analysed the P100 of the ERP. Children with blindness showed earlier latencies for cognitive (perceptual) event related potentials, shorter reaction times, and (paradoxically) worse ability to identify the spatial direction of the stimulus. On the other hand, they are equally proficient in recognizing stimuli with semantic content (letters). The last observation is consistent with the role of P100 on somatosensory-based recognition of complex forms. The cortical differences between seeing control and blind groups, during spatial tactile discrimination, are associated with activation in visual pathway (occipital) and task-related association (temporal and frontal) areas. The present results show that early processing of tactile stimulation conveying cross modal information differs in children with blindness or with normal vision.

## Introduction

Simultaneous multi-modal brain processing of spatial information is frequent [1, 2]. When the prepotent channels, visual and auditive, are faulty or overloaded, tactile information may be used to supply relevant data [3, 4, 5, 6]. This is the case in people with blindness. Tactile object recognition is associated with occipital (visual) cortex activation; specifically, the lateral occipital cortex (LOC), an area initially thought of as specialized in visual recognition of objects, but also activated by tactile recognition [7, 8, 9, 10, 11, 12]. Therefore this area is an example of multi-modal brain processing of spatial information [13, 14, 15, 16].

Neurophysiology is an excellent tool to study multi-modal processing given its excellent temporal resolution, much better than standard neuroimaging techniques such as fMRI, PET, etc. Mapping the exact temporal sequence of cortical activation after object presentation is paramount to separate mandatory involvement from spurious associations. More specifically, quantitative electroencephalography (qEEG) is a relatively simple and low-cost technique to approach this problem as its temporal resolution is in the range of msec. Neuroplasticity related to multi-modal adaptation to blindness, as well as the temporal sequence of hetero-modal cortical activation, can be studied with event related potentials (ERP) [17, 18].

Neuroplasticity is a process by which neurons change their connectivity in a stable fashion as a result of experience, learning, and sensory and cognitive stimulation [3, 15]. The potential neuroplasticity may be retained even while the pathway is deprived from its corresponding stimulation [5]. Neuroplasticity is an ongoing process. Sensory deprivation in different life stages is an optimal window to observe and characterize substitutive neuroplasticity in visual cortical areas of people with blindness [6, 7, 19]. Substitutive neuroplasticity is maximal at the beginning [8], during childhood and early adolescence [20]. In congenital blindness, cortical reorganization also displays cross-modal features [3, 7, 19, 21].

Cross modality can be defined as the brain capacity to process and interpret a given stimulus in a sensory modality different than the input one or, more generically, a perception which implies interactions between two or more sensory modalities [3]. Visual pathways in particular seem to process sensory information regardless of the sensory modality input [19]. A supra-modal concept of cortical functional organization is emerging which relies at least in part on cognitive processing of sensory information.

Compared to their seeing counterparts, people with blindness have a greater tactile capacity [22]. Having had a previous visual experience may confer some advantage in tactile processing to people with blindness [23]. Somato-sensory ERP provide temporal information on cortical processing of spatial information delivered through tact [24, 25]. Circa 50–100 msec, the activity is predominantly contralateral and in the primary sensory cortex [25, 26], around 100 msec there is automatic recognition of forms and shapes in somato-sensory areas [10]. There is already activation of secondary somato-sensory cortex from 100 msec onwards and bi-laterality happens from 150 msec onwards [25]. Differences in the physiology of object recognition between people with blindness and seeing people have been well documented [4, 11, 12. 27]. Furthermore, tactile recognition of letters, other than Braille, in people with blindness, is a very little studied field [12, 28]. Yet, graphesthetic ability may differ from random or less organized tactile stimulation in that it requires semantic processing and therefore should engage additional cortical processes and compel attention.

We now use a passive vibrotactile device to focus on the differences in spatial brain processing in children with blindness vs. normally seeing children, when learning a simple spatial task (lines with different orientations) or a task involving recognition of letters, to describe the early stages of its temporal sequence (from 80 to 220 msec) and to search for evidence of multi-modal cortical organization. We propose to test the hypothesis that blind children are able to remap tactile stimulation to visual cortical areas when it contains spatial information, whereas normally seeing children do not remap the input.

## Material and Methods

### Sample

We studied 24 children, 12 with blindness (5 girls and 7 boys) and 12 sighted (6 girls and 6 boys). All children were 8 and 11 years old, and had similar IQ, schooling level, and cultural characteristics. Causes of blindness are summarized in Table 1. Voluntary participants were chosen among students from 12 randomly selected schools serving children with blindness from the Madrid area. Written information about the experiment was provided to each school. After principals and teachers approved the research protocol, a formal talk was organized in each school and parents and teachers were provided detailed information (verbally and in writing) about the nature and purpose of the experiment. Interested parents provided written consent following individual informative sessions. Children were allowed to ask questions in the context of group and individual sessions, and all participants provided assent.

**Table 1:** Demographic Characteristics of Children with Blindness.

	Age	Sex	age of onset	light perception	etiology
**1**	8	Female	Congenital	OD no	retinal dystrophy
				OS no	
**2**	8	Male	Congenital	OD yes	optic atrophy
				OS yes	
**3**	9	Male	Congenital	OD yes	optic disc coloboma
				OS yes	
**4**	9	Male	Congenital	OD yes	retinal dystrophy
				OS yes	
**5**	9	Female	Congenital	OD yes	optic atrophy
				OS yes	
**6**	9	Male	Congenital	OD yes	retinal dystrophy
				OS yes	
**7**	11	Female	3 years	OD yes	optic atrophy
				OS yes	
**8**	11	Male	Congenital	OD yes	retinal dystrophy
				OS yes	
**9**	9	female	Congenital	OD no	Anophtalmia
				OS no	
**10**	10	female	Congenital	OD yes	retinal dystrophy
				OS yes	
**11**	8	male	6 years	OD yes	retinal dystrophy
				OS yes	
**12**	10	male	1 year	OD yes	optic atrophy
				OS yes	

Inclusion criteria for the participants were (a) age between 8 and 11, (b) active schooling and, (c) normal IQ. The IQ was verified from the psychological school reports. Exclusion criteria were: (a) having another sensorial deficit different than blindness, (b) present or past neuropsychiatric disease and, (d) history of obstetric trauma with cerebral hypoxia. The research protocol was approved by the Ethical Committee of the Hospital Clínico Universitario San Carlos (Madrid) and was in full compliance with the Declaration of Helsinki.

### Tactile Stimulation

#### The tactile stimulation system is composed of two elements

**Fig 1:**
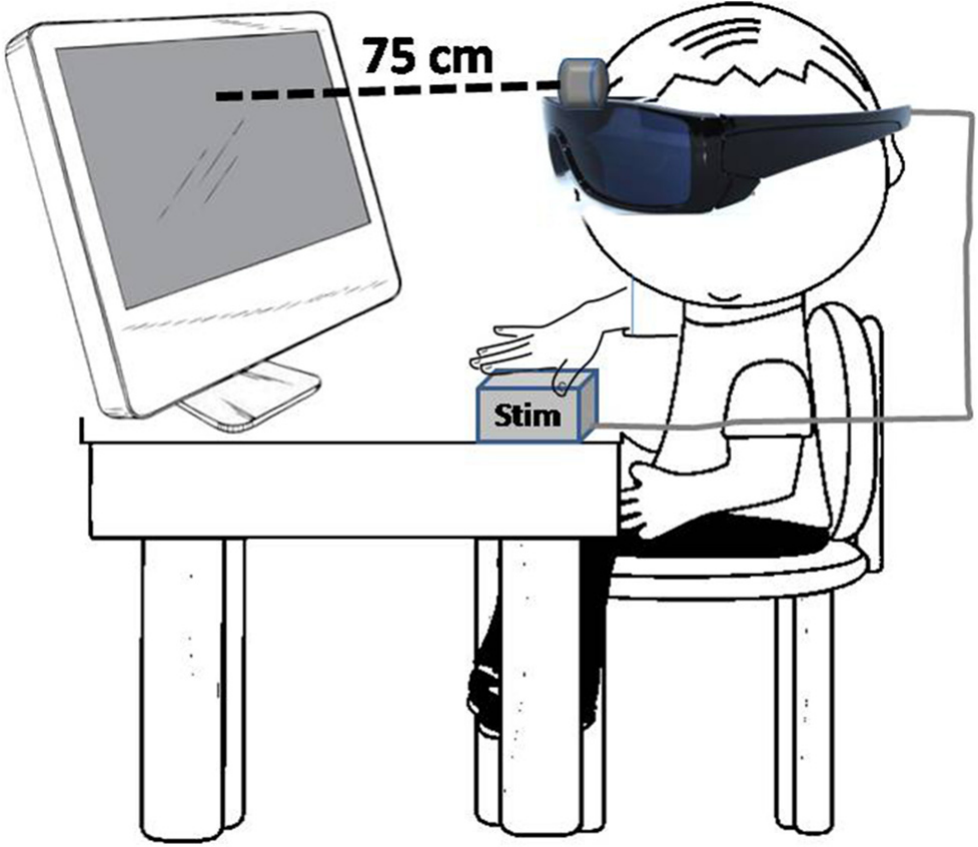
Schematic representation of the stimulus presentation set up. Stimuli are flashed in the LCD screen, read out by a camera mounted on the dark glasses, transformed into digital input and fed as tactile stimulation to the hand of the subject.

a micro-camera (visual receptor) and a tactile stimulator (stimulation matrix). The former is mounted on an eyeglass frame and the latter is passively touched by the volunteer. Images from the surrounding environment are captured by the micro-camera and transferred to the tactile stimulator either wirelessly or through a cable. The stimulator has a microprocessor inside, equipped with *ad hoc* algorithms which transform images captured by the micro-camera into vibro-tactile impulses. The stimulation matrix has 28X28 stimulation points, corresponding to binned pixels of the image captured by the micro-camera. The stimulation matrix is passively touched by the child with blindness with his/her left hand. Images projected on a flat screen and captured by the camera occupying the whole field of view presented at a rate of one per second. Half of the stimuli were lines and half were letters. The experiment was carried out in a very dimly lit room isolated from external noise. Subjects sat in an armchair, 75 cm in front of a 19” LCD screen (refresh rate 100 Hz) that displayed the stimuli (see schematic representation on Fig 1), and were provided with a keyboard to enter responses to recognized shapes. They were asked to be as relaxed as possible. The stimuli were delivered to seeing children exactly as they were presented to the non-seeing children, using the same set up with dark glasses, such that they did not see the screen but received the same tactile stimuli. Two tasks were performed.

#### Lines orientation task

300 lines stimuli were presented, 80% of the lines were oriented vertically, and 20% horizontaly; with a random order of presentation. Stimulus duration in the centre of the screen was 300 msec, followed by a black screen interval of 700 msec. Total duration of the lines task was 5 min. For event related potentials the low frequency stimulus (horizontal line) was designed as target, and a motor response (press the space bar with their right hand) was required whenever it appeared on the screen.

#### Letters recognition task

300 stimuli, either a letter “L” or an “N”, presented respectively in 80% and 20% of the occasions, following a pseudorandom sequence. Duration of each letter in the centre of the screen was 300 msec, followed by a black screen interval of 700 msec. The total duration of the letters task was 5 min. Once again, the low frequency stimulus (“N”) was designed as the target, and a motor response (press the space bar with their right hand) was required whenever it appeared on the screen.

### Electrophysiology

**Fig 2:**
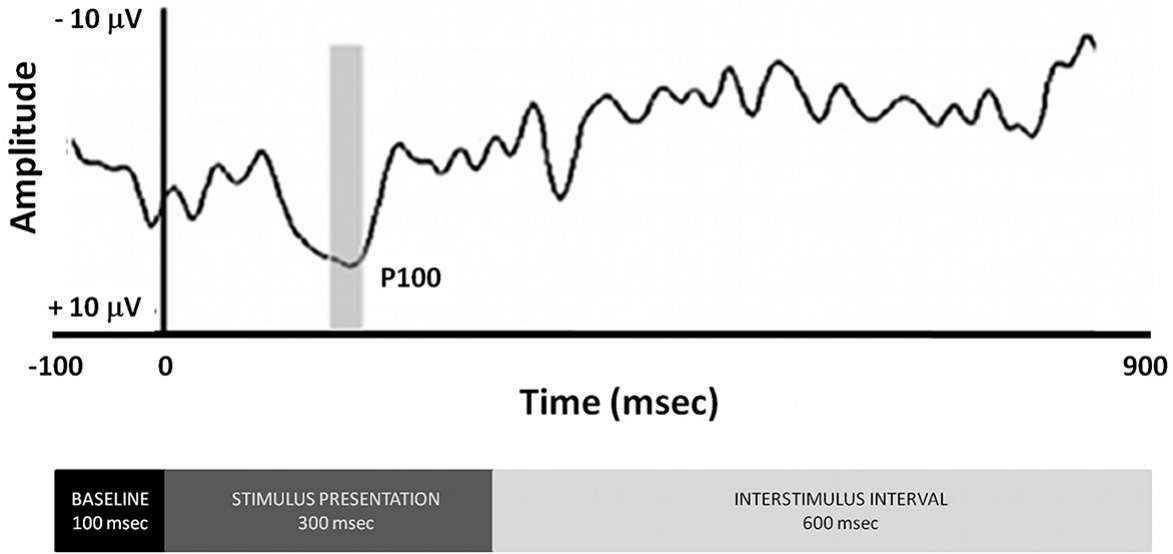
Event Related Potentials Following presentation of tactile presentation of spatial information. Time frame to analyze the P100 component was 80–220 ms and it was determined by searching for the maximal amplitude in the respective time window at the Pz electrode. The BMA analysis was made opening a time window of -20 to +20 ms starting from the high amplitude pick measured in Pz electrode. The bottom of the figure displays the time blocks of the experimental design.

High-density (128 channel) EEG recordings were obtained during tactile stimulation using a custom-designed electrode Neuroscan cap and an ATI EEG system (Advantek SRL, Argentina). Impedances were kept under 5kΩ. Additional channels were included to monitor eye movement (right and left lateral-canthi and superior and inferior orbits of the left eye) and for references (bilateral mastoids). Data were processed to an average reference following acquisition with a band-pass filter of 0.05–30 Hz and a sample rate of 512 Hz. An artefact rejection criterion of 100 mV was used to exclude eye blinks. Individual subject averages were visually inspected to insure that clean recordings were obtained. Eye and muscle movement artefacts were identified off-line on a trial-by-trial basis through visual inspection, and they were removed prior to data averaging and ERP analysis. Eye movement and muscle contraction artifacts detection was achieved by direct visual inspection of the EEG waves prior to any analysis. We used three electrodes placed to help identify eye and lid movements, namely cantus, supraciliar, and inferior palpebral. Upon inspection of these electrodes, intrinsic eye movements and blinking contamination of the EEG signal was selected and manually marked. Following detection the duration of the artifact prototype is established by accurately marking the beginning and end of the artifact event. We used the “Minimum variance of the data subspace” option on the PCA variance used as an estimate of brain activity by the analysis software. We retained all PCA topographies that explain at least a minimal variance of 5 to 10%. A second set of parameters for the PCA components that explain the artifact subspace allows selecting the number of those components that will be used for the correction. We included in the analysis components exhibiting 95% or more of the accumulated spectral power. In the case of eye blinks and movements, the first component is usually able to explain more than 99% of the total variance, especially if the artifact prototype was correctly identified. Typically more components have to be included to correct cardiac or muscle artifacts. Noisy channels were sparingly replaced with linear interpolations from clean channels. From the remaining artifact-free trials (mean = 215, range 187–232), averages were computed for each participant and each condition. Epochs were 1000 msec in duration (100 msec pre-stimulus, 900 msec post-stimulus, inclusive of the 300 msec stimulus), see Fig 1. Baseline was defined as the average voltage over the period of 100 ms prior to stimulus onset. EEG analysis was carried out on frequent (non-target) trials to avoid contamination by motor-related neural activity associated with making a response. ERPs obtained were averaged separately for each condition and each subject. We analysed the P100 component generated 80–220 msec after the trigger. The Pz electrode was used to measure the latency of the ERP. Once the ERP latency is determined at Pz, a window of +/- 20 msec is then chosen to localize the sources in all electrodes. Source localization analyses were based on greatest positive inflexion registered in the Pz electrode between 80 and 220 msec (11). Fig 2 describes the temporal evolution of the somatosensory evoked potential in relation to the experimental design (bottom). The gray bar shows the analysis window for P100 wave, the most prominent response at the Pz. The BMA analysis was made opening a time window of -20 to +20 msec starting from the highest amplitude peak measured in Pz electrode between 80–220 msec time window.

### Source Localization Reconstruction

The sources of the P100 component were estimated from 123 electrode recordings in the 24 subjects. The sources of the ERP are localized through the solution ofthe EEG inverse problem using the Bayesian Model Averaging (BMA) approach [29], and individual models were solved with Low Resoloution Electromagnetic Tomography (LORETA) [30]. Each model was defined by constraining the solution to a particular anatomical structure or combination of them using the SPM8 software package (The MathWorks, Natick, MA); the cerebellum (18 areas) and 8 areas that comprised less than 10 voxels were excluded from consideration.

Primary current density (PCD) was estimated, using the BMA method, for each subject’s P100 component for each of the three sessions. SPM was used to make population-level inferences over the calculated sources of the P100. Subsequently, SPMs were computed based on a voxel-by-voxel independent Hotelling T2 test against zero [31] between groups to estimate statistically significant sources for lines and letters. The resulting probability maps were thresholded at a false discovery rate (FDR) q ¼ 0.05 [32] and were depicted as 3D activation images overlaid on the MNI average brain [33]. Anatomical structures with cluster size greater than 10 voxels surviving the threshold were identified, and local maxima were measured and located according to MNI coordinates system.

### Statistics

For the analysis of individual latencies, group means were computed from individual event related potentials (mean = 215 individual stimuli per subject per condition). We also averaged reaction times (out of 60 individual stimuli per subject per condition) to shape recognition of the stimulus, and computed percent correct identifications. To understand if the behavioral and physiological parameters studied were able to correctly distinguish children with blindness from their seeing counterparts, a canonical discriminant analysis was performed, with 1000 iteration of bootstrapping, and cross validation, using the corresponding routines from SPSS 22 (IBM SPSS Statistics, Armonk, NY).

## Results

### Behavioural measurements

Blind subjects were significantly less able to identify the line orientation than the seeing controls, and their reaction times were significantly shorter (Table 2). One the other hand, whereas the blind subjects also had shorter reaction times to identify letters, their ability to identify them correctly was indistinguishable from that of seeing children (Table 2).

**Table 2:** Behavioral Performance on Tactile Recognition Tasks. Values provided represent mean and standard deviations for reaction times, successes and errors on line orientation and letter recognition tasks.

		Seeing	Blind	ANOVA F	*p =*
**Lines**	P100 latency	158.9 ± 26.3	104.8 ± 9.6	44.7	***0*.*0000***
	RT	855.1 ± 40.9	705.9 ± 127.3	14.9	***0*.*0008***
	Correct	39.5 ± 11	22.5 ± 9.9	15.9	***0*.*0006***
	Errors	17.1 ± 8.1	30.8 ± 16	7.0	***0*.*0150***
**Letters**	P100 latency	167.6 ± 22.3	101.8 ± 21.2	54.6	***0*.*0000***
	RT	796.2 ± 51.5	642.7 ± 126.2	15.2	***0*.*0008***
	Correct	19.4 ± 15.3	20.2 ± 7.7	0.0	*0*.*8811*
	Errors	29.9 ± 13	40.3 ± 23.7	1.8	*0*.*1948*

### Event related potentials (ERP) latencies

When individual latencies were averaged across subjects and blocked by stimulus type (lines vs letters), we found that blind children had significantly shorter latencies than seeing children for both stimuli types (Table 3). However, P100 latencies did not significantly differ by stimulus type (lines vs letters) in either group of children.

**Table 3:** Latency for P100 Evoked Potential Responses following presentation of lines or letters in non-target trials. Values represent mean and standard deviations of the P100 latency.

Group				95% Confidence Interval	
	Task	P100 latency	Std. Error	Lower Bound	Upper Bound
**Blind**	**Lines**	104.8	6.0	92.7	117.0
	**Letters**	101.8	6.0	89.7	114.0
**Seeing**	**Lines**	158.9	6.0	146.8	171.0
	**Letters**	167.6	6.0	155.5	179.7

### Classification of Samples

A canonical discriminant analysis with P100 latencies, reaction times, errors and correct responses for lines and for letters was performed. A single discriminant function was extracted, accounting for 100.0% of the variance (Χ^2^ = 35.037, p = 0.000). Remarkably, after cross validation only one subject was misclassified (a child with blindness was predicted to be seeing). Full results are provided in S1 Table.

### Event Related Potentials

**Fig 3:**
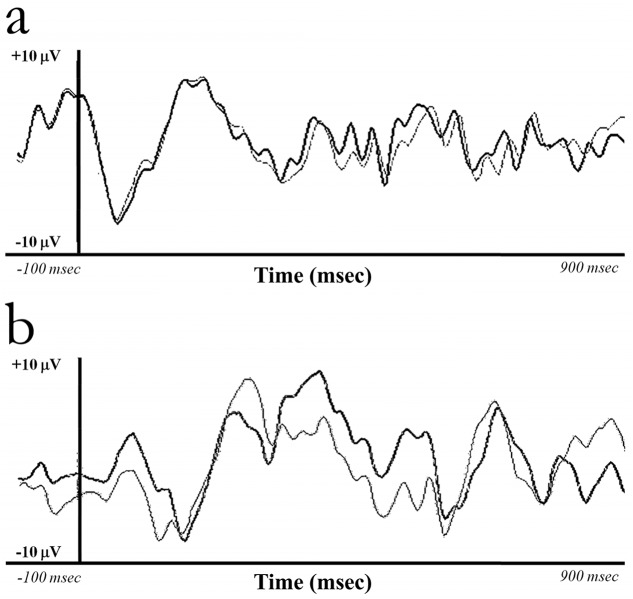
Grand average of Event Related Potentials in children with blindness (thick line) and their seeing counterparts (thin lines) following presentation of non-target stimuli on the line orientation (panel a) and letter recognition (panel b) tasks. The time frames for analysis of the P100 component were determined by searching for the maximal amplitude in the respective time window at the Pz electrode between 80–220 ms.

Fig 3 describes the temporal evolution of the somatosensory evoked potential. In response to tactile lines (Fig 3, panel a) and letters (Fig 3, panel b) stimuli, the cerebral evoked potentials in both groups were associated with the first positive wave (P100) of the event related potential. The configurations of these waves were similar for the cerebral responses in children with blindness and in the seeing controls.

### Source Localization

**Fig 4:**
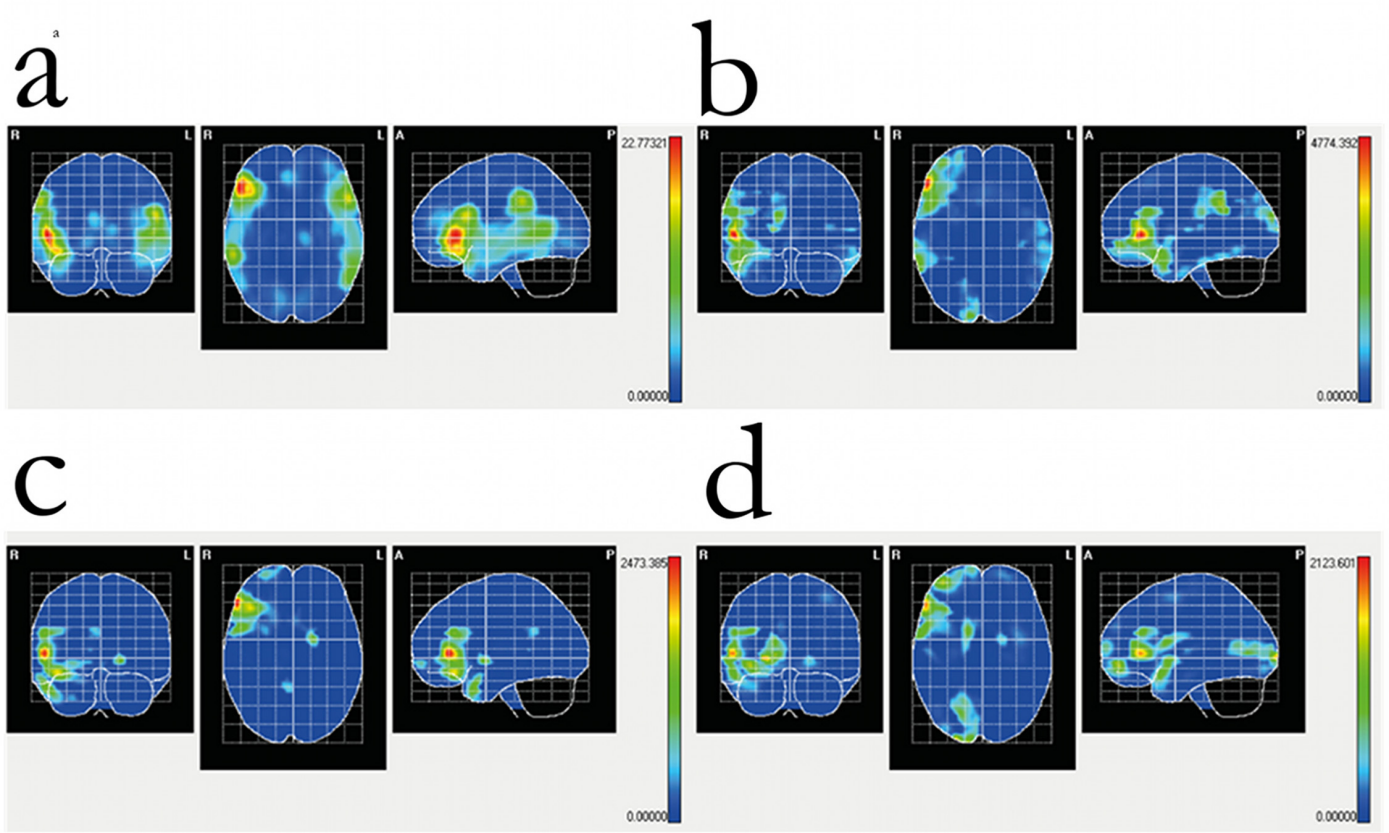
Cortical intensity projection (BMA) mean maps obtained at the P100 windows following non-target stimuli presentation in the line orientation and letter recognition tasks. Maximal intensity projection areas are displayed in red. Panel a: letter recognition in seeing children. Panel b: letter recognition in blind children. Panel c: line orientation in seeing children. Panel d: line orientation in blind children. Maximal intensity projection areas are displayed in red and yelow.

During the tactile recognition of letters (non-target trials), areas of maximal activation in children with blindness were located in the following cortical areas: right Frontal Inferior, pars orbitalis and pars triangularis; and right Supramarginal. In the control group (seeing children) maximal activity was localized in the following cortical areas: left Frontal Inferior, pars orbitalis, bilateral Frontal Inferior, pars triangularis, and right Supramarginal. During recognition of line orientation (non-target trials), maximal activity in children with blindness was located to the following cortical areas: right Calcarine; right Frontal Inferior, pars triangularis; right Frontal Middle; and right Temporal Pole, superior; as well as in the right Caudate. In control (seeing) children, the following cortical areas were activated during the same task: right Frontal Inflerior, pars orbitalis and pars triangularis; and right Temporal Pole, Middle (Fig 4).

### Differences in Source localization between children with normal sight and with blindness

**Fig 5:**
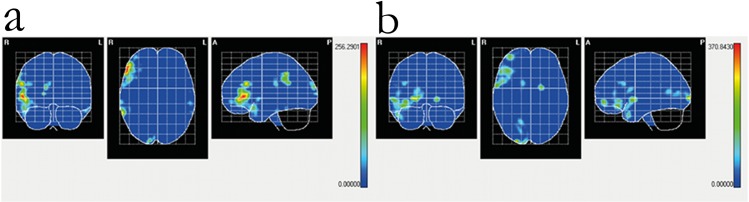
Statistical Mapping (SM) independent Hotelling T2 significant differences in P100 peak intensity between children with blindness and their seeing controls following presentation of non-target stimuli in the letter recognition (panel a) and line orientation (panel b) tasks. Red color represents p < .05. For description of individual anatomical areas see Table 4.

At the peak amplitude of P100 during tactile presentation of lines, children with blindness had greater activation (compared to seeing children) in right occipital, inferior frontal and medial temporal cortex (see below), whereas seeing children had greater activation of right medial frontal and superior temporal cortex (Table 4). During the tactile presentation of letters, on the other hand, children with blindness had greater P100 activations in right frontal, supramarginal and temporal cortex (Table 4), as well as the left inferior temporal gyrus. In children with blindess, in summary, during tactile stimulation the areas of maximal activation during P100 were located in right visual and association areas regardless of the stimuli (Fig 4). Differences between the patterns of activation specific for each task are shown in statistical maps in Fig 5.

**Table 4:** Summary of significant differences between children with blindess and seeing controls at the maximal intensity projection areas for P100 during line or letter presentation (non-target trials). For each specific localization, independent Hotelling T2 significant differences between seeing controls and blind groups are provided. AAL = X, Y, Z = co-ordinates from MNI in three spatial axes. p< .05.

	Structure	Volume	T2	*p =*	x	y	z	
**Lines**	right Calcarine fissure and surrounding cortex	93	370.8	0.00017	26	-98	0	Blind > Control
	right Caudate nucleus	24	109.5	0.00000	14	10	16	Blind > Control
	right Inferior frontal gyrus, orbital part	89	168.0	0.00030	42	30	-16	Blind > Control
	right Inferior frontal gyrus, triangular part	47	110.8	0.00055	38	22	28	Blind > Control
	right Middle frontal gyrus, orbital part	38	199.5	0.00000	38	54	-8	Control > Blind
	right Temporal pole: middle temporal gyrus	28	136.4	0.00054	50	14	-32	Blind > Control
	right Temporal pole: superior temporal gyrus	46	208.3	0.00000	62	6	0	Control > Blind
**Letters**	right Cuneus	19	172.3	0.00000	18	-102	8	Blind > Control
	right Inferior frontal gyrus, orbital part	66	208.9	0.00003	50	42	-4	Blind > Control
	right Inferior frontal gyrus, triangular part	109	256.3	0.00028	54	38	0	Blind > Control
	right Middle frontal gyrus, orbital part	12	56.2	0.00000	38	54	-8	Blind > Control
	right Supramarginal gyrus	66	196.3	0.00001	62	-46	44	Blind > Control
	left Inferior temporal gyrus	18	58.0	0.00002	-58	-26	-24	Blind > Control
	right Temporal pole: middle temporal gyrus	14	67.7	0.00004	54	10	-24	Blind > Control
	right Temporal pole: superior temporal gyrus	42	105.0	0.00006	54	14	-16	Blind > Control

## Discussion

The present results show that early processing of tactile stimulation conveying cross modal information differs in children with blindness or with normal vision. Children with blindness show earlier latencies for cognitive (perceptual) event related potentials, shorter reaction times, and (paradoxically) worse ability to identify the spatial direction of the stimulus. On the other hand, they are equally proficient in recognizing stimuli with semantic content (letters). The last observation is consistent with the role of P100 on somatosensory-based recognition of complex forms [34]. The cortical differences between seeing control and blind groups, during spatial tactile discrimination, are associated with activation in visual pathway (occipital) and task-related association (temporal and frontal) areas.

Why do children with blindness have shorter P100 latencies? P100 is negatively modulated by attention such that increased attention leads to longer latency [35]. Seeing children pressumably are less reliant on tact to identify shapes, and therefore need to put more effort into the task. This would also be consistent with the longer reaction times of seeing children. The fact P100 latency is decreased in children with blindness suggests a more automatic process, requiring less effortful attention.

The specific areas activated during P100 are parts of the visual pathways (calcarine sulcus, cuneus, inferior temporal gyrus), binding of complex percepts (superior temporal gyrus and the middle temporal gyrus), mutimodal perceptual integration (supramarginal gyrus), and the cognitive component of the tasks (inferior and middle frontal gyrus).

The right laterality of maximally activated areas during P100 deserves discussion. Automatic (non-target) processing of lines and letters led to widespread activation or right hemisphere structures, as expected given the spatial nature of the stimuli. What is perhaps more surprising is that maximal activation of secondary somatosensory cortex was ipsilateral to the stimulation. Similar findings have been reported with unilateral manual stimulation [36, 37, 38], along with some contradictory findings [1, 33]. However, none of the previous studies was carried out in children, and thus our result may reflect a specific developmental effect.

Tactile object recognition has been shown to involve visual systems, pressumably to access an internal object representation, as well as frontal polar structures subserving visuospatial working memory [39]. In humans, a key element in this pathway includes Brodmann areas 19 and 37, commonly referred to as lateral occipital complex (LOC), which is robustly and consistently activated by somatosensory haptic recognition, particularly for objects which also have a visual representation [2, 13, 14, 15, 16, 34]. However, the same structure is strongly activated during somatosensory object recognition in people with blindess, suggesting that visual imagery is not an obligatory condition for tactile object recognition in visual cortex [3, 4, 21, 40] and others. Our findings confirm that tactile object recognition involves this pathway in children with blindness as well. Electrophysiological studies show that engagement of visual cortex in tactile object recognition occurs early [24, 34, 41], usually between 100–150 msec post-trigger; our data are consistent with that description for the lines orientation task, but not for letters (~250 msec).

Both tactile recognition tasks reported in this manuscript resulted in activation of the right Brodmann area 38 in the temporal pole (rostral portions of the superior temporal gyrus and the middle temporal gyrus) (Table 4), a region probably involved in binding complex, highly processed perceptual inputs to visceral emotional responses [42]. Children with blindness probably undergo a more intense activation because their investment in "seeing" through the device (that is, they were instructed prior to the experiment that the stimulus on their hand was fed from a video camera). On the other hand, only the letter-recognition task resulted in more intense activation (in children with blindess) of the left inferior temporal gyrus (Table 4); this structure represents one of the higher levels of the ventral stream of visual processing, associated with the representation of complex object features, such as global shape (namely visual object recognition) or perception of faces and recognition of numbers [43]. Since this region is understood to be responsible for producing the “what” from visual input, it is likely that in children with blindness it takes over the assignation for haptic recognition.

Both tasks resulted in greater activation of the right inferior frontal gyrus and the right middle frontal gyrus in children with blindness. The former of these structures includes Brodmann area 45 (pars triangularis), and Brodmann area 47 (pars orbitalis), and in the right hemisphere is involved in processing of go/no go tasks [44], just as reported here. The latter (right middle frontal gyrus) is a probabilistic area (defined by the atlas) roughly corresponding to area 46 in the dorsolateral prefrontal cortex, the major brain structure responsible for sustaining attention and working memory, as well as self-control. In adults with congenital blindness, contrasted responses to a vibrotactile one-back frequency retention task with high working memory demand heavily engaged the right hemisphere dorsolateral prefrontal cortex [45], and as confirmed in the present study, adults with normal vision had significantly less activation. This may be due to the higher deman for attention resources in blind [34, 45].

The second structure selectively engaged by the letter discrimination task (along with the left inferior temporal gyrus) was the supramarginal gyrus, corresponding to Brodmann area 40. In the left hemisphere, this structure is engage by language perception and processing. However, it has specific functions in somatosensory shape discrimination [46, 47]. In patients with a selective visual deficit, but without any tactile defect, the sight of touch improves the visual impairment and this effect is associated with a lesion to the supramarginal gyrus [48]. Furthermore, inhibition with transcranial magnetic stimulation over the right supramarginal gyrus induced a bias shift in line discrimination in near space [49]. Notably, it has been shown that the left hemisphere predominates during encoding wheras the right hemisphere predominates during the matching [50], as is the case in the data reported here. However, adults with blindness have more extensive bilateral activity in supramarginal gyrus during recognition of Braile letters [51].

We previously reported that a similar pattern of activation during haptic recognition in subjects with blindness became eventually associated with a subjective experience of "*seeing*"[11]; however, the children with blindness reported here did not experience visual qualia. A possible explanation for this difference is that increasing the variety and number of stimuli, as we did with the adult sample, may result in plasticity in the visual cortex mediated through stimulus-selective response potentiation [52]. Alternatively, studies in congenitally blind and bilaterally enucleated individuals show that an early loss of sensory driven activity can lead to massive functional reorganization resulting in aberrant patterns of thalamocortical and corticocortical connections which could account for the development of visual qualia from tactile input, wheras the maintenance of normal patterns of connections in the absence of visual input suggests may impose severe constraints precluding such adaptations [53].

## Supporting Information

S1 Table Full results of a canonical discriminant analysis using as variables the reaction times to recognition of lines and letters (LineasRT, LetrasRT), % of correct responses for lines and letters (ALineas, ALetras), % errors for lines and letters (ELineas, ELetras), and p100 latency for lines and letters (LI1p100, LE1p100). The discriminant groups were blind (1) and seeing (2) children. A single discriminant function was found (Chi square = 35.037; df = 8; p< .000). For the original data, squared Mahalanobis distance is based on canonical functions. 100% of original cases were correctly classified. For the cross-validated data, squared Mahalanobis distance is based on observations. In cross validation, each case is classified by the functions derived from all cases other than that case. 95.8% of cross-validated grouped cases correctly classified.
(HTM)
