# Supplementary material for: Differences in Early Stages of Tactile ERP Temporal Sequence (P100) in Cortical Organization during Passive Tactile Stimulation in Children with Blindness and Controls
Source: PLoS One. 2015 Jul 30;10(7):e0124527. doi: 10.1371/journal.pone.0124527 (PMC4520520; doi:10.1371/journal.pone.0124527)
Supplement: S1 Table — The discriminant groups were blind (1) and seeing (2) children. A single discriminant function was found (Chi square = 35.037; df = 8; p< .000). For the original data, squared Mahalanobis distance is based on canonical functions. 100% of original cases were correctly classified. For the cross-validated data, squared Mahalanobis distance is based on observations. In cross validation, each case is classified by the functions derived from all cases other than that case. 95.8% of cross-validated grouped cases correctly classified. (HTM) [file pone.0124527.s001.htm]

| Canonical Discriminant Analysis |  |  |  |  |  |  |  |  |  |  |  |
|  |  |  |  |  |  |  |  |  |  |  |  |
| Notes | | |  |  |  |  |  |  |  |  |  |
| Output Created | | 13-JAN-2014 16:24:57 |  |  |  |  |  |  |  |  |  |
| Comments | |  |  |  |  |  |  |  |  |  |  |
| Input | Data |  |  |  |  |  |  |  |  |  |  |
| Active Dataset | DataSet0 |  |  |  |  |  |  |  |  |  |
| Filter | <none> |  |  |  |  |  |  |  |  |  |
| Weight | <none> |  |  |  |  |  |  |  |  |  |
| Split File | <none> |  |  |  |  |  |  |  |  |  |
| N of Rows in Working Data File | 24 |  |  |  |  |  |  |  |  |  |
| Missing Value Handling | Definition of Missing | User-defined missing values are treated as missing in the analysis phase. |  |  |  |  |  |  |  |  |  |
| Cases Used | In the analysis phase, cases with no user- or system-missing values for any predictor variable are used. Cases with user-, system-missing, or out-of-range values for the grouping variable are always excluded. |  |  |  |  |  |  |  |  |  |
| Syntax | | DISCRIMINANT  � /GROUPS=Grupo(1 2)  � /VARIABLES=LI1p100 LineasRT ALineas ELineas LE1p100 LetrasRT ALetras ELetras  � /ANALYSIS ALL  � /PRIORS EQUAL  � /STATISTICS=MEAN STDDEV UNIVF BOXM COEFF CORR COV TCOV TABLE CROSSVALID  � /PLOT=COMBINED MAP  � /PLOT=CASES  � /CLASSIFY=NONMISSING POOLED. |  |  |  |  |  |  |  |  |  |
| Resources | Processor Time | 00:00:00.05 |  |  |  |  |  |  |  |  |  |
| Elapsed Time | 00:00:00.20 |  |  |  |  |  |  |  |  |  |
|  |  |  |  |  |  |  |  |  |  |  |  |
| [DataSet0] C:\Users\erausquing\Desktop\Ortiz Alonso ciegos\ciegos y control.sav |  |  |  |  |  |  |  |  |  |  |  |
|  |  |  |  |  |  |  |  |  |  |  |  |
| Warnings |  |  |  |  |  |  |  |  |  |  |  |
| No COMBINED plot is available for a single discriminant function. |  |  |  |  |  |  |  |  |  |  |  |
|  |  |  |  |  |  |  |  |  |  |  |  |
| Analysis Case Processing Summary | | | |  |  |  |  |  |  |  |  |
| Unweighted Cases | | N | Percent |  |  |  |  |  |  |  |  |
| Valid | | 24 | 100.0 |  |  |  |  |  |  |  |  |
| Excluded | Missing or out-of-range group codes | 0 | 0.0 |  |  |  |  |  |  |  |  |
| At least one missing discriminating variable | 0 | 0.0 |  |  |  |  |  |  |  |  |
| Both missing or out-of-range group codes and at least one missing discriminating variable | 0 | 0.0 |  |  |  |  |  |  |  |  |
| Total | 0 | 0.0 |  |  |  |  |  |  |  |  |
| Total | | 24 | 100.0 |  |  |  |  |  |  |  |  |
|  |  |  |  |  |  |  |  |  |  |  |  |
| Group Statistics | | | | | |  |  |  |  |  |  |
| Grupo | | Mean | Std. Deviation | Valid N (listwise) | |  |  |  |  |  |  |
| Unweighted | Weighted |  |  |  |  |  |  |
| 1.00 | LI1p100 | 104.8333 | 9.63736 | 12 | 12.000 |  |  |  |  |  |  |
| LineasRT | 705.9167 | 127.30454 | 12 | 12.000 |  |  |  |  |  |  |
| ALineas | 22.5000 | 9.88571 | 12 | 12.000 |  |  |  |  |  |  |
| ELineas | 30.7500 | 16.02909 | 12 | 12.000 |  |  |  |  |  |  |
| LE1p100 | 101.8333 | 21.22962 | 12 | 12.000 |  |  |  |  |  |  |
| LetrasRT | 642.6667 | 126.23379 | 12 | 12.000 |  |  |  |  |  |  |
| ALetras | 20.1667 | 7.69691 | 12 | 12.000 |  |  |  |  |  |  |
| ELetras | 40.3333 | 23.65023 | 12 | 12.000 |  |  |  |  |  |  |
| 2.00 | LI1p100 | 158.9167 | 26.32130 | 12 | 12.000 |  |  |  |  |  |  |
| LineasRT | 855.1667 | 40.92306 | 12 | 12.000 |  |  |  |  |  |  |
| ALineas | 39.5833 | 11.04913 | 12 | 12.000 |  |  |  |  |  |  |
| ELineas | 17.0833 | 8.06179 | 12 | 12.000 |  |  |  |  |  |  |
| LE1p100 | 167.5833 | 22.34628 | 12 | 12.000 |  |  |  |  |  |  |
| LetrasRT | 796.1667 | 51.47256 | 12 | 12.000 |  |  |  |  |  |  |
| ALetras | 19.4167 | 15.34724 | 12 | 12.000 |  |  |  |  |  |  |
| ELetras | 29.9167 | 12.99271 | 12 | 12.000 |  |  |  |  |  |  |
| Total | LI1p100 | 131.8750 | 33.74626 | 24 | 24.000 |  |  |  |  |  |  |
| LineasRT | 780.5417 | 119.84518 | 24 | 24.000 |  |  |  |  |  |  |
| ALineas | 31.0417 | 13.46325 | 24 | 24.000 |  |  |  |  |  |  |
| ELineas | 23.9167 | 14.23687 | 24 | 24.000 |  |  |  |  |  |  |
| LE1p100 | 134.7083 | 39.77598 | 24 | 24.000 |  |  |  |  |  |  |
| LetrasRT | 719.4167 | 122.61673 | 24 | 24.000 |  |  |  |  |  |  |
| ALetras | 19.7917 | 11.87976 | 24 | 24.000 |  |  |  |  |  |  |
| ELetras | 35.1250 | 19.40487 | 24 | 24.000 |  |  |  |  |  |  |
|  |  |  |  |  |  |  |  |  |  |  |  |
| Tests of Equality of Group Means | | | | | |  |  |  |  |  |  |
|  | Wilks' Lambda | F | df1 | df2 | Sig. |  |  |  |  |  |  |
| LI1p100 | .330 | 44.674 | 1 | 22 | .000 |  |  |  |  |  |  |
| LineasRT | .595 | 14.949 | 1 | 22 | .001 |  |  |  |  |  |  |
| ALineas | .580 | 15.932 | 1 | 22 | .001 |  |  |  |  |  |  |
| ELineas | .760 | 6.962 | 1 | 22 | .015 |  |  |  |  |  |  |
| LE1p100 | .287 | 54.604 | 1 | 22 | .000 |  |  |  |  |  |  |
| LetrasRT | .591 | 15.214 | 1 | 22 | .001 |  |  |  |  |  |  |
| ALetras | .999 | .023 | 1 | 22 | .881 |  |  |  |  |  |  |
| ELetras | .925 | 1.788 | 1 | 22 | .195 |  |  |  |  |  |  |
|  |  |  |  |  |  |  |  |  |  |  |  |
| Pooled Within-Groups Matricesa | | | | | | | | | |  |  |
|  | | LI1p100 | LineasRT | ALineas | ELineas | LE1p100 | LetrasRT | ALetras | ELetras |  |  |
| Covariance | LI1p100 | 392.845 | 331.455 | -44.337 | 22.890 | 221.557 | 66.705 | 26.307 | 94.845 |  |  |
| LineasRT | 331.455 | 8940.572 | 134.652 | -355.019 | -631.833 | 7939.561 | -138.167 | -789.886 |  |  |
| ALineas | -44.337 | 134.652 | 109.905 | -87.140 | -14.140 | 260.538 | 41.504 | -74.928 |  |  |
| ELineas | 22.890 | -355.019 | -87.140 | 160.962 | -.367 | -461.553 | -23.223 | 90.413 |  |  |
| LE1p100 | 221.557 | -631.833 | -14.140 | -.367 | 475.027 | -332.583 | 52.428 | 100.102 |  |  |
| LetrasRT | 66.705 | 7939.561 | 260.538 | -461.553 | -332.583 | 9292.197 | 34.129 | -949.614 |  |  |
| ALetras | 26.307 | -138.167 | 41.504 | -23.223 | 52.428 | 34.129 | 147.390 | -73.557 |  |  |
| ELetras | 94.845 | -789.886 | -74.928 | 90.413 | 100.102 | -949.614 | -73.557 | 364.072 |  |  |
| Correlation | LI1p100 | 1.000 | .177 | -.213 | .091 | .513 | .035 | .109 | .251 |  |  |
| LineasRT | .177 | 1.000 | .136 | -.296 | -.307 | .871 | -.120 | -.438 |  |  |
| ALineas | -.213 | .136 | 1.000 | -.655 | -.062 | .258 | .326 | -.375 |  |  |
| ELineas | .091 | -.296 | -.655 | 1.000 | -.001 | -.377 | -.151 | .373 |  |  |
| LE1p100 | .513 | -.307 | -.062 | -.001 | 1.000 | -.158 | .198 | .241 |  |  |
| LetrasRT | .035 | .871 | .258 | -.377 | -.158 | 1.000 | .029 | -.516 |  |  |
| ALetras | .109 | -.120 | .326 | -.151 | .198 | .029 | 1.000 | -.318 |  |  |
| ELetras | .251 | -.438 | -.375 | .373 | .241 | -.516 | -.318 | 1.000 |  |  |
| a. The covariance matrix has 22 degrees of freedom. | | | | | | | | | |  |  |
|  |  |  |  |  |  |  |  |  |  |  |  |
| Covariance Matricesa | | | | | | | | | |  |  |
| Grupo | | LI1p100 | LineasRT | ALineas | ELineas | LE1p100 | LetrasRT | ALetras | ELetras |  |  |
| Total | LI1p100 | 1138.810 | 2422.766 | 198.614 | -170.924 | 1139.571 | 2229.489 | 14.582 | -56.245 |  |  |
| LineasRT | 2422.766 | 14362.868 | 793.933 | -871.692 | 1955.600 | 13570.851 | -161.361 | -1161.114 |  |  |
| ALineas | 198.614 | 793.933 | 181.259 | -144.257 | 279.491 | 933.286 | 36.357 | -118.092 |  |  |
| ELineas | -170.924 | -871.692 | -144.257 | 202.688 | -234.764 | -988.746 | -19.540 | 123.620 |  |  |
| LE1p100 | 1139.571 | 1955.600 | 279.491 | -234.764 | 1582.129 | 2314.736 | 37.284 | -82.918 |  |  |
| LetrasRT | 2229.489 | 13570.851 | 933.286 | -988.746 | 2314.736 | 15034.862 | 2.612 | -1325.446 |  |  |
| ALetras | 14.582 | -161.361 | 36.357 | -19.540 | 37.284 | 2.612 | 141.129 | -68.321 |  |  |
| ELetras | -56.245 | -1161.114 | -118.092 | 123.620 | -82.918 | -1325.446 | -68.321 | 376.549 |  |  |
| a. The total covariance matrix has 23 degrees of freedom. | | | | | | | | | |  |  |
|  |  |  |  |  |  |  |  |  |  |  |  |
| Analysis 1 |  |  |  |  |  |  |  |  |  |  |  |
| Box's Test of Equality of Covariance Matrices |  |  |  |  |  |  |  |  |  |  |  |
|  |  |  |  |  |  |  |  |  |  |  |  |
| Log Determinants | | |  |  |  |  |  |  |  |  |  |
| Grupo | Rank | Log Determinant |  |  |  |  |  |  |  |  |  |
| 1.00 | 8 | 43.878 |  |  |  |  |  |  |  |  |  |
| 2.00 | 8 | 38.675 |  |  |  |  |  |  |  |  |  |
| Pooled within-groups | 8 | 46.907 |  |  |  |  |  |  |  |  |  |
| The ranks and natural logarithms of determinants printed are those of the group covariance matrices. | | |  |  |  |  |  |  |  |  |  |
|  |  |  |  |  |  |  |  |  |  |  |  |
| Test Results | | |  |  |  |  |  |  |  |  |  |
| Box's M | | 123.855 |  |  |  |  |  |  |  |  |  |
| F | Approx. | 2.052 |  |  |  |  |  |  |  |  |  |
| df1 | 36 |  |  |  |  |  |  |  |  |  |
| df2 | 1628.589 |  |  |  |  |  |  |  |  |  |
| Sig. | .000 |  |  |  |  |  |  |  |  |  |
| Tests null hypothesis of equal population covariance matrices. | | |  |  |  |  |  |  |  |  |  |
|  |  |  |  |  |  |  |  |  |  |  |  |
| Summary of Canonical Discriminant Functions |  |  |  |  |  |  |  |  |  |  |  |
| Eigenvalues | | | | |  |  |  |  |  |  |  |
| Function | Eigenvalue | % of Variance | Cumulative % | Canonical Correlation |  |  |  |  |  |  |  |
| 1 | 6.004a | 100.0 | 100.0 | .926 |  |  |  |  |  |  |  |
| a. First 1 canonical discriminant functions were used in the analysis. | | | | |  |  |  |  |  |  |  |
|  |  |  |  |  |  |  |  |  |  |  |  |
| Wilks' Lambda | | | | |  |  |  |  |  |  |  |
| Test of Function(s) | Wilks' Lambda | Chi-square | df | Sig. |  |  |  |  |  |  |  |
| 1 | .143 | 35.037 | 8 | .000 |  |  |  |  |  |  |  |
|  |  |  |  |  |  |  |  |  |  |  |  |
| Standardized Canonical Discriminant Function Coefficients | |  |  |  |  |  |  |  |  |  |  |
|  | Function |  |  |  |  |  |  |  |  |  |  |
| 1 |  |  |  |  |  |  |  |  |  |  |
| LI1p100 | .294 |  |  |  |  |  |  |  |  |  |  |
| LineasRT | .538 |  |  |  |  |  |  |  |  |  |  |
| ALineas | .670 |  |  |  |  |  |  |  |  |  |  |
| ELineas | .302 |  |  |  |  |  |  |  |  |  |  |
| LE1p100 | .798 |  |  |  |  |  |  |  |  |  |  |
| LetrasRT | -.173 |  |  |  |  |  |  |  |  |  |  |
| ALetras | -.376 |  |  |  |  |  |  |  |  |  |  |
| ELetras | -.217 |  |  |  |  |  |  |  |  |  |  |
|  |  |  |  |  |  |  |  |  |  |  |  |
| Structure Matrix | |  |  |  |  |  |  |  |  |  |  |
|  | Function |  |  |  |  |  |  |  |  |  |  |
| 1 |  |  |  |  |  |  |  |  |  |  |
| LE1p100 | .643 |  |  |  |  |  |  |  |  |  |  |
| LI1p100 | .582 |  |  |  |  |  |  |  |  |  |  |
| ALineas | .347 |  |  |  |  |  |  |  |  |  |  |
| LetrasRT | .339 |  |  |  |  |  |  |  |  |  |  |
| LineasRT | .336 |  |  |  |  |  |  |  |  |  |  |
| ELineas | -.230 |  |  |  |  |  |  |  |  |  |  |
| ELetras | -.116 |  |  |  |  |  |  |  |  |  |  |
| ALetras | -.013 |  |  |  |  |  |  |  |  |  |  |
| Pooled within-groups correlations between discriminating variables and standardized canonical discriminant functions   �Variables ordered by absolute size of correlation within function. | |  |  |  |  |  |  |  |  |  |  |
|  |  |  |  |  |  |  |  |  |  |  |  |
| Functions at Group Centroids | |  |  |  |  |  |  |  |  |  |  |
| Grupo | Function |  |  |  |  |  |  |  |  |  |  |
| 1 |  |  |  |  |  |  |  |  |  |  |
| 1.00 | -2.346 |  |  |  |  |  |  |  |  |  |  |
| 2.00 | 2.346 |  |  |  |  |  |  |  |  |  |  |
| Unstandardized canonical discriminant functions evaluated at group means | |  |  |  |  |  |  |  |  |  |  |
|  |  |  |  |  |  |  |  |  |  |  |  |
| Classification Statistics |  |  |  |  |  |  |  |  |  |  |  |
| Classification Processing Summary | | |  |  |  |  |  |  |  |  |  |
| Processed | | 24 |  |  |  |  |  |  |  |  |  |
| Excluded | Missing or out-of-range group codes | 0 |  |  |  |  |  |  |  |  |  |
| At least one missing discriminating variable | 0 |  |  |  |  |  |  |  |  |  |
| Used in Output | | 24 |  |  |  |  |  |  |  |  |  |
|  |  |  |  |  |  |  |  |  |  |  |  |
| Prior Probabilities for Groups | | | |  |  |  |  |  |  |  |  |
| Grupo | Prior | Cases Used in Analysis | |  |  |  |  |  |  |  |  |
| Unweighted | Weighted |  |  |  |  |  |  |  |  |
| 1.00 | .500 | 12 | 12.000 |  |  |  |  |  |  |  |  |
| 2.00 | .500 | 12 | 12.000 |  |  |  |  |  |  |  |  |
| Total | 1.000 | 24 | 24.000 |  |  |  |  |  |  |  |  |
|  |  |  |  |  |  |  |  |  |  |  |  |
| Classification Function Coefficients | | |  |  |  |  |  |  |  |  |  |
|  | Grupo | |  |  |  |  |  |  |  |  |  |
| 1.00 | 2.00 |  |  |  |  |  |  |  |  |  |
| LI1p100 | -.342 | -.273 |  |  |  |  |  |  |  |  |  |
| LineasRT | .248 | .275 |  |  |  |  |  |  |  |  |  |
| ALineas | .831 | 1.130 |  |  |  |  |  |  |  |  |  |
| ELineas | .871 | .983 |  |  |  |  |  |  |  |  |  |
| LE1p100 | .547 | .719 |  |  |  |  |  |  |  |  |  |
| LetrasRT | -.053 | -.061 |  |  |  |  |  |  |  |  |  |
| ALetras | .393 | .248 |  |  |  |  |  |  |  |  |  |
| ELetras | .484 | .431 |  |  |  |  |  |  |  |  |  |
| (Constant) | -117.692 | -172.029 |  |  |  |  |  |  |  |  |  |
| Fisher's linear discriminant functions | | |  |  |  |  |  |  |  |  |  |
| Casewise Statistics | | | | | | | | | | | |
| Case Number | | Actual Group | Highest Group | | | | | Second Highest Group | | | Discriminant Scores |
| Predicted Group | P(D>d | G=g) | | P(G=g | D=d) | Squared Mahalanobis Distance to Centroid | Group | P(G=g | D=d) | Squared Mahalanobis Distance to Centroid | Function 1 |
| p | df |
| Original | 1 | 1 | 1 | .548 | 1 | 1.000 | .362 | 2 | .000 | 16.734 | -1.745 |
| 2 | 1 | 1 | .954 | 1 | 1.000 | .003 | 2 | .000 | 22.557 | -2.403 |
| 3 | 1 | 1 | .089 | 1 | 1.000 | 2.887 | 2 | .000 | 40.849 | -4.045 |
| 4 | 1 | 1 | .848 | 1 | 1.000 | .037 | 2 | .000 | 23.855 | -2.538 |
| 5 | 1 | 1 | .652 | 1 | 1.000 | .204 | 2 | .000 | 26.454 | -2.797 |
| 6 | 1 | 1 | .917 | 1 | 1.000 | .011 | 2 | .000 | 21.045 | -2.241 |
| 7 | 1 | 1 | .584 | 1 | 1.000 | .300 | 2 | .000 | 17.177 | -1.798 |
| 8 | 1 | 1 | .919 | 1 | 1.000 | .010 | 2 | .000 | 21.073 | -2.244 |
| 9 | 1 | 1 | .489 | 1 | 1.000 | .479 | 2 | .000 | 15.999 | -1.654 |
| 10 | 1 | 1 | .324 | 1 | .998 | .972 | 2 | .002 | 13.736 | -1.360 |
| 11 | 1 | 1 | .246 | 1 | 1.000 | 1.346 | 2 | .000 | 34.251 | -3.506 |
| 12 | 1 | 1 | .598 | 1 | 1.000 | .278 | 2 | .000 | 17.348 | -1.819 |
| 13 | 2 | 2 | .227 | 1 | .995 | 1.459 | 1 | .005 | 12.141 | 1.138 |
| 14 | 2 | 2 | .072 | 1 | 1.000 | 3.232 | 1 | .000 | 42.118 | 4.144 |
| 15 | 2 | 2 | .751 | 1 | 1.000 | .100 | 1 | .000 | 19.142 | 2.029 |
| 16 | 2 | 2 | .265 | 1 | .997 | 1.244 | 1 | .003 | 12.794 | 1.231 |
| 17 | 2 | 2 | .163 | 1 | 1.000 | 1.947 | 1 | .000 | 37.057 | 3.741 |
| 18 | 2 | 2 | .811 | 1 | 1.000 | .057 | 1 | .000 | 19.827 | 2.107 |
| 19 | 2 | 2 | .598 | 1 | 1.000 | .278 | 1 | .000 | 27.243 | 2.873 |
| 20 | 2 | 2 | .751 | 1 | 1.000 | .100 | 1 | .000 | 19.142 | 2.029 |
| 21 | 2 | 2 | .319 | 1 | .998 | .993 | 1 | .002 | 13.658 | 1.350 |
| 22 | 2 | 2 | .045 | 1 | 1.000 | 4.033 | 1 | .000 | 44.895 | 4.354 |
| 23 | 2 | 2 | .207 | 1 | .994 | 1.592 | 1 | .006 | 11.767 | 1.084 |
| 24 | 2 | 2 | .784 | 1 | 1.000 | .075 | 1 | .000 | 19.516 | 2.072 |
| Cross-validatedb | 1 | 1 | 1 | .451 | 8 | .999 | 7.825 | 2 | .001 | 20.869 |  |
| 2 | 1 | 1 | .388 | 8 | 1.000 | 8.478 | 2 | .000 | 28.848 |  |
| 3 | 1 | 1 | .000 | 8 | 1.000 | 27.922 | 2 | .000 | 84.926 |  |
| 4 | 1 | 1 | .241 | 8 | 1.000 | 10.359 | 2 | .000 | 32.273 |  |
| 5 | 1 | 1 | .887 | 8 | 1.000 | 3.657 | 2 | .000 | 29.021 |  |
| 6 | 1 | 1 | .000 | 8 | .997 | 40.853 | 2 | .003 | 52.767 |  |
| 7 | 1 | 1 | .340 | 8 | .999 | 9.029 | 2 | .001 | 22.199 |  |
| 8 | 1 | 1 | .000 | 8 | .959 | 69.342 | 2 | .041 | 75.661 |  |
| 9 | 1 | 1 | .064 | 8 | .990 | 14.769 | 2 | .010 | 24.049 |  |
| 10 | 1 | 1 | .245 | 8 | .982 | 10.296 | 2 | .018 | 18.335 |  |
| 11 | 1 | 1 | .011 | 8 | 1.000 | 19.752 | 2 | .000 | 59.581 |  |
| 12 | 1 | 1 | .012 | 8 | .992 | 19.683 | 2 | .008 | 29.317 |  |
| 13 | 2 | 1\*\* | .004 | 8 | .804 | 22.883 | 2 | .196 | 25.707 |  |
| 14 | 2 | 2 | .000 | 8 | 1.000 | 58.217 | 1 | .000 | 138.886 |  |
| 15 | 2 | 2 | .006 | 8 | .998 | 21.287 | 1 | .002 | 33.627 |  |
| 16 | 2 | 2 | .715 | 8 | .991 | 5.390 | 1 | .009 | 14.805 |  |
| 17 | 2 | 2 | .144 | 8 | 1.000 | 12.157 | 1 | .000 | 53.467 |  |
| 18 | 2 | 2 | .816 | 8 | 1.000 | 4.438 | 1 | .000 | 22.256 |  |
| 19 | 2 | 2 | .742 | 8 | 1.000 | 5.148 | 1 | .000 | 31.482 |  |
| 20 | 2 | 2 | .976 | 8 | 1.000 | 2.159 | 1 | .000 | 19.838 |  |
| 21 | 2 | 2 | .658 | 8 | .994 | 5.905 | 1 | .006 | 16.052 |  |
| 22 | 2 | 2 | .052 | 8 | 1.000 | 15.389 | 1 | .000 | 71.045 |  |
| 23 | 2 | 2 | .269 | 8 | .943 | 9.942 | 1 | .057 | 15.540 |  |
| 24 | 2 | 2 | .294 | 8 | 1.000 | 9.608 | 1 | .000 | 25.712 |  |
| For the original data, squared Mahalanobis distance is based on canonical functions.  �For the cross-validated data, squared Mahalanobis distance is based on observations. | | | | | | | | | | | |
| b. Cross validation is done only for those cases in the analysis. In cross validation, each case is classified by the functions derived from all cases other than that case. | | | | | | | | | | | |
|  |  |  |  |  |  |  |  |  |  |  |  |
| Classification Resultsa,c | | | | | |  |  |  |  |  |  |
| Grupo | | | Predicted Group Membership | | Total |  |  |  |  |  |  |
| 1.00 | 2.00 |  |  |  |  |  |  |
| Original | Count | 1.00 | 12 | 0 | 12 |  |  |  |  |  |  |
| 2.00 | 0 | 12 | 12 |  |  |  |  |  |  |
| % | 1.00 | 100.0 | 0.0 | 100.0 |  |  |  |  |  |  |
| 2.00 | 0.0 | 100.0 | 100.0 |  |  |  |  |  |  |
| Cross-validatedb | Count | 1.00 | 12 | 0 | 12 |  |  |  |  |  |  |
| 2.00 | 1 | 11 | 12 |  |  |  |  |  |  |
| % | 1.00 | 100.0 | 0.0 | 100.0 |  |  |  |  |  |  |
| 2.00 | 8.3 | 91.7 | 100.0 |  |  |  |  |  |  |
| a. 100.0% of original grouped cases correctly classified. | | | | | |  |  |  |  |  |  |
| b. Cross validation is done only for those cases in the analysis. In cross validation, each case is classified by the functions derived from all cases other than that case. | | | | | |  |  |  |  |  |  |
| c. 95.8% of cross-validated grouped cases correctly classified. | | | | | |  |  |  |  |  |  |
|  |  |  |  |  |  |  |  |  |  |  |  |
|  |  |  |  |  |  |  |  |  |  |  |  |
|  |  |  |  |  |  |  |  |  |  |  |  |
